# Temporal trends in sudden infant death syndrome in Canada from 1991 to 2005: contribution of changes in cause of death assignment practices and in maternal and infant characteristics

**DOI:** 10.1111/j.1365-3016.2011.01248.x

**Published:** 2012-03

**Authors:** Nicolas L Gilbert, Deshayne B Fell, K S Joseph, Shiliang Liu, Juan Andrés León, Reg Sauve

**Affiliations:** 1 Maternal and Infant Health SectionPublic Health Agency of Canada; 2 Better Outcomes Registry & Network (BORN) OntarioOttawa; 3 Department of Obstetrics and Gynaecology and the School of Population and Public Health, University of British Columbia and the Children's and Women's Hospital of British ColumbiaVancouver; 4 Department of Community Health Sciences, University of CalgaryCalgary, Canada

**Keywords:** SIDS, time trend, Canada

## Abstract

Gilbert NL, Fell DB, Joseph KS, Liu S, León JA, Sauve R, for the Fetal and Infant Health Study Group of the Canadian Perinatal Surveillance System. Temporal trends in sudden infant death syndrome in Canada from 1991 to 2005: contribution of changes in cause of death assignment practices and in maternal and infant characteristics. *Paediatric and Perinatal Epidemiology* 2012; **26:** 124–130.

The rate of sudden infant death syndrome (SIDS) declined significantly in Canada and the US between the late 1980s and the early 2000s. In the US, this decline was shown to be due in part to a shift in diagnosis, as deaths from accidental suffocation and strangulation in bed and from other ill-defined and unspecified cause increased concurrently. This study was undertaken to determine whether there was such a shift in diagnosis from SIDS to other causes of death in Canada, and to quantify the true temporal decrease in SIDS. Cause-specific infant death rates were compared across three periods: 1991–95, 1996–2000 and 2001–05 using the Canadian linked livebirth-infant death file. The temporal decline in SIDS was estimated after adjustment for maternal and infant characteristics such as maternal age and small-for-gestational age using logistic regression. Deaths from SIDS decreased from 78.4 [95% confidence interval (CI) 73.4, 83.4] per 100 000 livebirths in 1991–95, to 48.5 [95% CI 44.3, 52.7] in 1996–2000 and to 34.6 [95% CI 31.0, 38.3] in 2001–05. Mortality rates from other ill-defined and unspecified causes and accidental suffocation and strangulation in bed remained stable. The temporal decline in SIDS between 1991–95 and 2001–05 did not change substantially after adjustment for maternal and infant factors. It is unlikely that the temporal decline of SIDS in Canada was due to changes in cause-of-death assignment practices or in maternal and infant characteristics.

## Introduction

Sudden infant death syndrome (SIDS) is defined as ‘the sudden death of an infant aged less than one year, which remains unexplained after a thorough case investigation, including performance of a complete autopsy, examination of the death scene, and review of the clinical history.’^1^ Rates of SIDS have been declining in Canada and the US since the late 1980s.^2^,^3^ In Canada (excluding Ontario), the rate of SIDS decreased from 0.6 to 0.3 deaths per 1000 livebirths between 1999 and 2004.^4^ Nevertheless, SIDS remains the fourth leading cause of infant death – in 2004, SIDS accounted for 5.0% of all infant deaths in Canada (excluding Ontario).^4^

Several explanations have been proposed for this decline in SIDS. These include parents' compliance with public health recommendations to reduce the risk of SIDS, in particular the advice that infants sleep on their back.^5^–^7^ Another risk factor for SIDS, maternal smoking during pregnancy,^8^,^9^ has also declined steadily in the past decades,^4^ while breast feeding, which is known to have a protective effect,^10^ has increased markedly.^4^

More recently, it has been postulated that the observed decline in SIDS was partly artefactual, that is, due to a shift in diagnosis or reporting practices of physicians certifying deaths.^11^ An analysis of death records in the US provided support for this hypothesis: between 1995–98 and 1999–2001 mortality from SIDS declined from 77.4 to 60.8 per 100 000 livebirths, a difference of 16.6 per 100 000. During the same period, deaths from other ill-defined and unspecified causes, accidental suffocation and strangulation in bed, and other accidental suffocation and strangulation rose by approximately 6.2, 3.4, and 0.7 per 100 000 livebirths, respectively, for a total increase of 10.3 per 100 000.^12^

In recent years, there have been significant changes in the distribution of certain characteristics of mothers and infants. Between 1995 and 2004, the proportions of women who gave birth at 35–39 and 40–49 years of age in Canada increased from 9.8% to 12.6% and from 1.4% to 2.6%, respectively.^4^ During the same period, the proportion of babies born small-for-gestational age (SGA) decreased from 10.1% to 7.8%.^4^ These changes may have been partly responsible for the decline in SIDS as older maternal age and SGA are associated with a decreased and an increased risk of SIDS, respectively.^2^

This study was undertaken to investigate temporal trends in SIDS in Canada between 1991 and 2005. We attempted to determine whether temporal changes in rates of SIDS, other ill-defined and unspecified cause, and accidental suffocation and strangulation in bed supported the hypothesis that the decline in SIDS was partly due to a change in diagnosis and reporting practices. We also examined whether changes in maternal and infant characteristics such as maternal age, parity, SGA and other factors could explain any part of the decline in SIDS.

## Methods

The data source was the Canadian linked livebirth-infant death file created through the probabilistic linking of livebirth and death registrations by Statistics Canada.^13^ Livebirths from Ontario were excluded because of previously documented concerns related to the completeness of birth registrations in that province.^4^ Cause-specific and overall death rates per 100 000 livebirths were calculated by birth cohort year and 5-year period (1991–95, 1996–2000 and 2001–05). The causes of death codes studied included SIDS and other causes that were found to increase in the US as SIDS decreased^12^ (namely, accidental suffocation and strangulation in bed, other suffocation and strangulation, other ill-defined and unspecified cause, and abandonment/maltreatment, and asphyxia and respiratory arrest). However, rates per specific year were reported only for SIDS and for the sum of accidental suffocation and strangulation in bed and other ill-defined and unspecified cause (in order to avoid reporting cell sizes smaller than five). Absolute differences and relative differences in rates (with their accompanying binomial 95% confidence intervals) were calculated comparing 1996–2000 with 1991–95, and 2001–05 with 1996–2000.

To determine if the temporal decrease in SIDS was confounded by changes in maternal and infant characteristics, unadjusted and adjusted odds ratios expressing period effects were estimated using logistic regression. This analysis was restricted to singleton livebirths for simplicity and because of a lack of consensus on SGA cut-off for multiple births. As SIDS was a very rare event, odds ratios for this outcome are approximately equal to relative risks. Demographic factors known to be associated with the risk of SIDS were included as covariates: maternal age, parity, sex, gestational age and being SGA. Infants were classified as being SGA if their birthweight-for-gestational age was below the 10th percentile of the Canadian reference value.^14^

Finally, the proportion of the change in SIDS rates attributable to changes in maternal and infant characteristics was evaluated using the Kitagawa standardisation and decomposition method^15^ as modified by Yang *et al*.^16^ Briefly, all singleton livebirths were stratified into 18 groups formed by any combination of maternal age (<20, 20–29 and ≥30 years), parity (0, 1 or 2+) and SGA (yes or no). Unadjusted and standardised rates of SIDS were calculated for each group for 1991–95, 1996–2000 and 2001–05 birth periods. Rates were standardised for maternal age, parity and SGA using 1991–95 as the reference period. The change in standardised rates was the estimated change in SIDS rates that would have occurred if the distributions of maternal age, parity and SGA had not changed over time; the difference between unadjusted and standardised rates was the component of the decline in SIDS attributable to the change in these characteristics.

## Results

Between 1991–95 and 2001–05, the overall infant mortality rate declined by 18%, from 629 to 513 per 100 000 livebirths in Canada (Table 1). Most SIDS cases (93%) occurred during the post-neonatal period and the rate of SIDS declined by 56%, from 78.4 in 1991–95 to 34.6 per 100 000 livebirths in 2001–05. A larger proportion of the absolute decline occurred between the early and late 1990s. Other causes of death such as asphyxia and respiratory arrest, ill-defined and unspecified cause, accidental suffocation and strangulation and abandonment/maltreatment showed no change between 1991–95 and 2001–05, although other suffocation and strangulation increased from 1.2 to 2.4 per 100 000 livebirths (absolute increase 1.2 per 100 000 livebirths compared with an absolute decrease of 43.8 per 100 000 livebirths in SIDS; Table 1).

**Table 1:** Temporal trends in cause-specific infant mortality rates in Canada (excluding Ontario), 1991–2005

			Number of deaths			Deaths per 100 000 livebirths [95% confidence interval]			Change in mortality from 1991–95 to 2001–05	
					
Cause of death	ICD-9	ICD-10	1991–95	1996–2000	2001–05	1991–95	1996–2000	2001–05	Absolute change in rate (per 100 000 livebirths)	% change [95% confidence interval]
Sudden infant death syndrome	798.0	R95	945	512	352	78.4 [73.4, 83.4]	48.5 [44.3, 52.7]	34.6 [31.0, 38.3]	−43.8	−55.8 [−60.9, −50.1]
Asphyxia and respiratory arrest	799.0, 799.1	R09.0, R09.2	10	8	9	0.8 [0.3, 1.3]	0.8 [0.2, 1.3]	0.9 [0.3, 1.5]	+0.1	+6.7 [−56.6, 162.7]
Ill-defined/ unspecified cause	799.9	R99	163	161	137	13.5 [11.4, 15.6]	15.2 [12.9, 17.6]	13.5 [11.2, 15.7]	0.0	−0.3 [−20.6, 25.1]
Accidental suffocation/ strangulation in bed	E913.0	W75	24	17	19	2.0 [1.2, 2.8]	1.6 [0.8, 2.4]	1.9 [1.0, 2.7]	−0.1	−6.1 [−48.6, 71.4]
Other suffocation and strangulation	E913.1–E913.9	W76, W77, W81–W84	14	17	24	1.2 [0.6, 1.8]	1.6 [0.8, 2.4]	2.4 [1.4, 3.3]	+1.2	+103.3 [5.2, 293.0]
Abandonment/ maltreatment	E967, E968.4	Y06, Y07	19	17	9	1.6 [0.9, 2.3]	1.6 [0.8, 2.4]	0.9 [0.3, 1.5]	−0.7	−43.8 [−74.6, 24.2]

Total infant deaths			7579	5667	5215	628.8 [614.7, 642.9]	536.6 [522.7, 550.6]	513.1 [499.2, 527.0]	−115.7	−18.4 [−21.2, −15.5]

There were 1 205 375 livebirths in 1991–95, 1 056 038 in 1996–2000 and 1 016 374 in 2001–05.

ICD-9, the ninth revision of the International Classification of Diseases; ICD-10, the tenth revision of the International Classification of Diseases.

**Figure 1:**
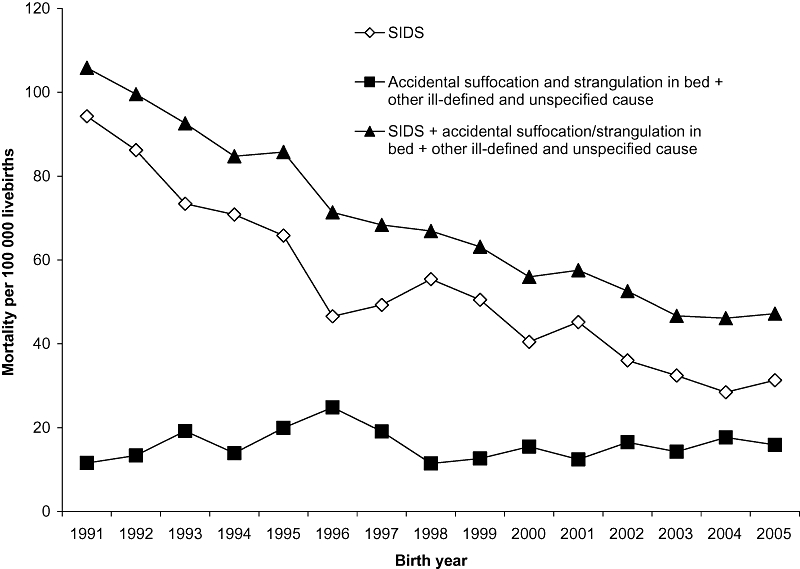
Rates of sudden infant death syndrome (SIDS), accidental suffocation and strangulation in bed, and unknown and unspecified causes in Canada (excluding Ontario), 1991–2005.

The examination of rates by individual birth year of SIDS, accidental suffocation and strangulation in bed and other ill-defined and unspecified cause (Figure 1) revealed an overall decline of SIDS by 63 per 100 000 livebirths and a slight increase of accidental suffocation and strangulation in bed and other ill-defined and unspecified cause (by 4 per 100 000) from 1991 to 2005. In 1996, there was a dip in the SIDS rates and a peak in the rate of other ill-defined and unspecified cause. Mortality due to these three causes of death combined declined steadily from 106 to 47 per 100 000 livebirths (Figure 1).

We observed a number of significant temporal trends in risk factors for SIDS over the study period – the proportion of older mothers (≥35 years), primiparous mothers, twins and preterm livebirths increased significantly between 1991–95 and 2001–05, while the proportion of SGA livebirths decreased (Table 2).

**Table 2:** Temporal changes in maternal and infant characteristics, Canada (excluding Ontario) 1991–2005

	% per birth cohort			
		
Determinant	1991–95 (*n* = 1 205 375)	1996–2000 (*n* = 1 056 0238)	2001–05 (*n* = 1 016 374)	*P*-value for linear trend
Maternal age ≥35 years	9.8	13.3	15.1	<0.001
Primiparity	42.5	43.2	44.0	<0.001
Multiple gestation	2.1	2.5	2.9	<0.001
Male sex	51.3	51.3	51.3	0.349
Preterm birth (<37 weeks)	6.7	7.3	7.8	<0.001
Small-for-gestational age	10.3	8.9	8.0	<0.001

Logistic regression analysis showed that temporal declines in SIDS among singleton livebirths (Table 3) were similar to those among all livebirths (Table 1). Younger maternal age, higher parity, multiple birth, preterm birth and SGA livebirth were associated with higher risk of SIDS. All these risk factors remained important factors in the multivariable logistic regression model. The temporal decline in SIDS did not change substantially after adjustment for these maternal and infant characteristics. A model excluding gestational age showed similar results (Table 3).

**Table 3:** Factors associated with sudden infant death syndrome (SIDS) in singleton infants, Canada (excluding Ontario) 1991–2005^a^

				Adjusted OR [95% CI]	
				
	Livebirths	SIDS	Unadjusted OR [95% CI]	Model 1^b^	Model 2^b^
Birth year					
1991–95	1 169 041	888	1.00 Reference	1.00 Reference	1.00 Reference
1996–2000	1 002 167	569	0.62 [0.55, 0.69]	0.65 [0.58, 0.73]	0.65 [0.58, 0.73]
2001–05	970 174	316	0.43 [0.38, 0.49]	0.48 [0.43, 0.55]	0.49 [0.43, 0.56]
Maternal age (years)					
10–19	194 388	323	2.81 [2.48, 3.18]	4.32 [3.78, 4.95]	4.46 [3.89, 5.11]
20–29	1 681 098	996	1.00 Reference	1.00 Reference	1.00 Reference
30–34	874 230	250	0.48 [0.420, 0.55]	0.39 [0.34, 0.45]	0.38 [0.33, 0.44]
≥35	391 666	104	0.45 [0.37, 0.55]	0.30 [0.25, 0.37]	0.30 [0.25, 0.37]
Parity					
0	1 374 304	495	1.00 Reference	1.00 Reference	1.00 Reference
1	1 088 031	540	1.38 [1.22, 1.56]	2.17 [1.91, 2.46]	2.15 [1.89, 2.44]
2	439 449	328	2.07 [1.80, 2.38]	3.81 [3.28, 4.42]	3.81 [3.29, 4.43]
3	147 971	173	3.25 [2.73, 3.86]	6.49 [5.41, 7.79]	6.61 [5.50, 7.93]
4+	91 627	137	4.16 [3.44, 5.02]	9.48 [7.75, 11.59]	9.85 [8.05, 12.05]
Sex					
Male	1 612 051	1024	1.50 [1.36, 1.65]	1.48 [1.34, 1.63]	1.50 [1.36, 1.65]
Female	1 529 331	649	1.00 Reference	1.00 Reference	1.00 Reference
Gestational age (weeks)^c^					
22–31	24 250	38	3.25 [2.36, 4.49]	2.86 [2.07, 3.95]	
32–36	165 386	202	2.53 [2.18, 2.93]	2.40 [2.07, 2.79]	
37–41	2 884 342	1393	1.00 Reference	1.00 Reference	
≥42	67 404	40	1.23 [0.90, 1.68]	1.02 [0.74, 1.39]	
Small-for-gestational age					
No	2 854 729	1410	1.00 Reference	1.00 Reference	1.00 Reference
Yes	286 653	263	1.86 [1.63, 2.12]	1.90 [1.67, 2.17]	1.89 [1.66, 2.16]

a Only records with complete information on maternal age, parity, gestational age, sex and birthweight were included. Multiple births and livebirths with a gestational age <22 weeks were also excluded.

b bMultiple logistic regression models including all variables for which odds ratios are shown.

c OR, odds ratio; CI, confidence interval.

The standardisation and decomposition analysis showed that 10.2% of the decrease of SIDS among singleton livebirths between 1991–95 and 2001–05 was attributable to temporal changes in maternal age, parity and SGA rates (Table 4).

**Table 4:** Temporal trends in sudden infant death syndrome (SIDS) rates in singleton infants, Canada, 1991–2005: standardisation and decomposition analysis^a^

				Change from 1991–95			Change from 1991–95		Change associated with maternal/infant factors	
							
Period	Number of livebirths^a^	Number of SIDS deaths	Crude rate of SIDS (per 100 000 livebirths)	Per 100 000 livebirths	% of 1991–95 value	Standardised rate of SIDS (per 100 000 livebirths)^b^	Per 100 000 livebirths	% of 1991–95 value	Per 100 000 livebirths	% of total variation
1991–95	1 169 041	888	76.0	0.0	0.0	76.0	0.0	0.0		
1996–2000	1 002 167	469	46.8	−29.2	−38.4	49.9	−26.1	−34.4	−3.1	10.5
2001–05	970 174	316	32.6	−43.4	−57.1	37.1	−39.0	−51.2	−4.4	10.2

a Only records with complete information on maternal age, parity, gestational age, sex and birthweight were included. Multiple births and livebirths with a gestational age <22 weeks were also excluded.

b Standardised for maternal age (<20, 20–29 and ≥30 years), parity (0, 1 or 2+) and small-for-gestational age (yes or no) based on the 1991–95 distribution.

## Discussion

Our study showed that the reduction in SIDS in Canada between 1981 and 1998^2^ continued in the 2000s and the rate in 2001–05 was approximately half of that observed in 1991–95. This is consistent with the overall decline in post-neonatal mortality observed in Canada between 1991 and 2004.^4^,^17^ Unlike the US, where the decline in SIDS rates between 1995–98 and 1999–2001 was offset by an increase in rates of death from ill-defined and unspecified causes and from accidental suffocation and strangulation in bed,^12^ there was no overall increase in these causes as SIDS declined in Canada. Shifting in diagnosis or reporting practices may have occurred, but this does not appear to explain a significant proportion of the SIDS decline.

The decrease in SIDS may be explained by a decreased prevalence of known risk factors among pregnant women and infants. Between 1994–95 and 2005, smoking rates in pregnant women declined from 23.5% to 13.4%, while the proportion of mothers who breast fed increased from 75% to 87%.^4^,^17^ In addition, 69% of Canadian parents placed their infants on their back for sleep in 2001, compared with 41% in 1999.^18^

Although changes over time in the frequency of older maternal age, multiparity and SGA infants reduced the proportion of infants at higher risk of SIDS, only one-tenth of the decrease in SIDS rates was found to be attributable to these changes. Moreover, an adjustment for these potential confounders did not substantially change the magnitude of the temporal reduction in the risk of SIDS.

This study had limitations including those associated with the use of death registries for epidemiological studies. Each jurisdiction in Canada (10 provinces and three territories) is responsible for vital event registration and regulation of medical practice, including requirement for autopsies and death reviews. Across all jurisdictions, infant deaths meeting the definition of SIDS require an autopsy, and in some jurisdictions, an additional review. However, if there is a delay between the death and the availability of autopsy results, the underlying cause of death information on the death registration record that is submitted to the national database may be based on the judgement of the physician who certified the death rather than autopsy findings.^19^ Probabilistic linkage of birth and death records can be subject to minor errors, but a validation study of the method indicated an accuracy of more than 99%.^13^

The underlying causes of death in Canadian death registrations were coded using the ninth revision of the International Classification of Diseases (ICD-9) up to 1999 and using the tenth revision (ICD-10) from 2000 onwards. This is unlikely to have affected SIDS itself as one ICD-9 code was replaced by one ICD-10 code with exactly the same descriptor. Other ill-defined and unspecified causes also retained the same descriptor. For ‘accidental suffocation and strangulation in bed’, there was a change, however, with ICD-9 referring to ‘accidental mechanical suffocation in bed or cradle’. Changes in inclusion or exclusion criteria of other conditions may have led to a change in the number of cases reported as other ill-defined and unspecified cause, for instance. However, the temporal pattern in death rates (Figure 1) showed no abrupt change in the causes of death under study. In addition, Statistics Canada investigated the comparability of ICD-9 and ICD-10 by dual-coding a random sample of deaths under both classifications. Comparability of classifications varied depending on causes of death, but was excellent for SIDS.^20^ Our study could not exclude a shift towards other causes of death that were not examined, for example, specific pathologies such as metabolic disorders that may have been better diagnosed in 2001–05 than in 1991–95.

The exclusion of Ontario, which represents approximately 38% of livebirths, potentially limits the generalisation of our results to all of Canada, but does not affect its internal validity for the provinces and territories included. Finally, we were unable to quantify the effect of changes in sleep position and maternal smoking because of a lack of information on these factors. However, this was not directly related to our study question about potential changes in diagnosis and coding of the underlying cause of death.

In conclusion, the rate of SIDS continued to decline in Canada in the 2000s. This study suggests that artefactual changes in diagnosis, changes in reporting practices for underlying causes of infant death and changes in the distribution of maternal or infant characteristics (such as maternal age, parity and SGA) are unlikely explanations for the temporal decline in SIDS in Canada.
